# Personalized Energy Expenditure Estimation: Visual Sensing Approach With Deep Learning

**DOI:** 10.2196/33606

**Published:** 2022-09-14

**Authors:** Toby Perrett, Alessandro Masullo, Dima Damen, Tilo Burghardt, Ian Craddock, Majid Mirmehdi

**Affiliations:** 1 University of Bristol Bristol United Kingdom

**Keywords:** energy expenditure, calories, calorimetry, deep learning, computer vision

## Abstract

**Background:**

Calorimetry is both expensive and obtrusive but provides the only way to accurately measure energy expenditure in daily living activities of any specific person, as different people can use different amounts of energy despite performing the same actions in the same manner. Deep learning video analysis techniques have traditionally required a lot of data to train; however, recent advances in few-shot learning, where only a few training examples are necessary, have made developing personalized models without a calorimeter a possibility.

**Objective:**

The primary aim of this study is to determine which activities are most well suited to calibrate a vision-based personalized deep learning calorie estimation system for daily living activities.

**Methods:**

The SPHERE (Sensor Platform for Healthcare in a Residential Environment) Calorie data set is used, which features 10 participants performing 11 daily living activities totaling 4.5 hours of footage. Calorimeter and video data are available for all recordings. A deep learning method is used to regress calorie predictions from video.

**Results:**

Models are personalized with 32 seconds from all 11 actions in the data set, and mean square error (MSE) is taken against a calorimeter ground truth. The best single action for calibration is *wipe* (1.40 MSE). The best pair of actions are *sweep* and *sit* (1.09 MSE). This compares favorably to using a whole 30-minute sequence containing 11 actions to calibrate (1.06 MSE).

**Conclusions:**

A vision-based deep learning energy expenditure estimation system for a wide range of daily living activities can be calibrated to a specific person with footage and calorimeter data from 32 seconds of sweeping and 32 seconds of sitting.

## Introduction

### Background

The ability to measure energy expenditure is important in a wide variety of settings. Examples range from sports training [1] to diabetes and cardiovascular disease monitoring [2]. Of particular interest is obesity management, where the amount of activity found in sedentary people at work and in the home can make a large difference to their overall fitness [3], especially when energy expenditure that is not due to exercise is taken into account [4]. The most accurate ways to measure person-specific energy expenditure are to use a sealed chamber [5] or indirect calorimetry [6]. However, other than the upfront costs and time with such equipment, they are also intrusive and cumbersome when used for a significant length of time, and they require expert installation. Further, they are unsuitable for long-term deployment in homes (eg, for health monitoring applications), whether for large scale studies or for individual cases.

In the absence of such accurate measurements, clinicians have used metabolic equivalent task (MET) tables [7,8] as an approximation, where each action has an associated energy expenditure value. This can be a time-consuming process, especially for a long sequence containing multiple activities, as each activity must be manually assigned start and end times. However, most importantly, METs are highly inaccurate compared to calorimetry. Hence, other approaches have sought to bridge the accuracy gap, while also reducing the burden on clinicians and annotators. For example, wearables have been explored as a cheaper, less intrusive, and more portable alternative [9-16] with improved results over METs. Large-scale home monitoring systems [17-19] have started to provide enough data to investigate computer vision approaches [20-22], which are cheap, much less intrusive, and more accurate. This provides the opportunity to extend the monitoring of energy consumption from stationary work environments [23-25], where variation between different people cannot be accurately captured by self-reporting.

The main problem with noncalorimeter-based approaches is that they still offer a *general model* only. That is, they will provide the same energy expenditure estimation for 2 individuals carrying out an action in a similar way, even though they may be using different amounts of energy.

Our aim is to estimate energy expenditure from observations of a person’s physical movement. To this end, we train a deep learning model using footage of participants wearing calorimeters. Traditionally, deep learning methods have required a vast amount of data to personalize [26,27]. However, we exploit recent advances that can adapt general models to specific tasks [28-32] and determine which small set of actions is best suited to personalizing a general model. This will reduce the amount of calorimeter time per participant necessary for model personalization and will demonstrate that vision-based deep learning models are suitable for use in real-world settings. This is the first time in the literature a personalized vision-based energy expenditure estimation training regime has been addressed. On a more fundamental level, determining which actions are most suitable for fine-tuning a deep neural network can also give an indication about which types of activity are necessary to indicate a person’s calorific profile. The approach introduced in this paper will be of practical use in many fields that monitor energy expenditure, such as sports training [1], nutrition [33], obesity management [34,35], and so on.

### Materials

For this study, we used the SPHERE (Sensor Platform for Healthcare in a Residential Environment) Calorie data set [36]. We briefly recap the key properties here before explaining our neural network approach to provide personalized energy expenditure estimations.

### Data Collection

A total of 10 participants performed a variety of daily living activities while using a K4b2 (COSMED) calorimeter. The activities consisted of the following: *stand*, *sit, walk*, *wipe*, *vacuum*, *sweep*, *lie*, *exercise*, *stretch*, *clean*, *and read*. The participants are filmed using an off-the-shelf RGB-D (Red, Green, Blue plus Depth) sensor, and the video footage is pseudonymized by extracting silhouettes [37]. In total, 4.5 hours of footage at 30 frames per second and calorimeter data are available. To obtain a ground truth label for each video frame, calorimeter data are interpolated between each breath reading.

## Methods

### Ethics Approval

No ethics approval was required for this study, as we only used publicly available anonymized data for the purpose it was designed for.

### Overview

In this section, we will use our recently developed deep learning method [30] and provide a brief overview. Deep learning models consist of a neural network architecture, which processes a data stream (to give the energy expenditure estimation in our case) with an associated training regime to adapt a randomly initialized model to the desired task—often referred to as a learned model.

### Architecture

**Figure 1:**
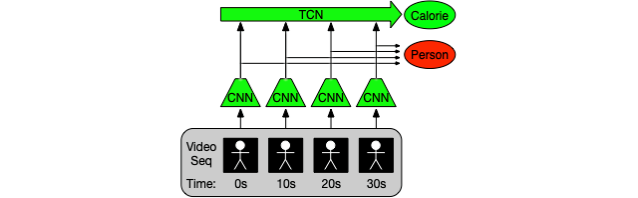
Neural network architecture for processing silhouette video streams, consisting of a convolutional neural network (CNN) for extracting frame features and a temporal convolutional network (TCN) for combining frame features over a period of 30 seconds. To achieve an initialization that can be quickly adapted to unseen participants, the main training objective is to minimize the calorie loss while maximizing the person loss. Seq: sequence.

Deep neural network video architectures typically consist of 2 subnetworks, which are as follows: (1) a spatial subnetwork to extract useful features from each video frame—this part is necessary as the type of action currently being performed and the participant's body position can be an indicator of how much energy they are consuming. This is the convolutional neural network (Figure 1). Specifically, ResNet-18 [38] with pretrained ImageNet [39] weights is used; and (2) a temporal subnetwork to combine features extracted from each frame and to use this information to make an estimation—this part is necessary because just using 1 video frame is insufficient for energy consumption estimation; how fast participants move as well as their previous behavior and actions can have a great effect and must therefore be considered. For this stage, we deploy a temporal convolutional network [40] (Figure 1).

These 2 subnetworks are trained jointly (in this paper, we refer to this combined architecture as the “network”), so they can learn to specialize short- or long-term observations effectively. Previous works have shown that around 30 seconds of video footage is required to accurately regress calorie values [21,36] as previous activity affects the current calorimeter reading. Thus, we take advantage of an architecture that uses the spatial subnetwork to observe at 1 frame per second and a temporal subnetwork to combine 30 seconds worth of spatial subnetwork features.

### Training

Given an architecture to process the video data, along with silhouette videos and calorimeter readings, a training regime is required to learn from examples in a training set. As a large amount of data is usually required to train a neural network [26,27], they are often “pretrained” on a related large data set, then “fine-tuned” on the data set being used. However, in the case of learning a personalized model, the data requirements are still too large to be used for conventional fine-tuning. Thus, we use our recently developed few-shot (otherwise known as “meta-learning” or “learning to learn”) technique [30], which aims to learn a model that can be fine-tuned with very little amount of data.

**Figure 2:**
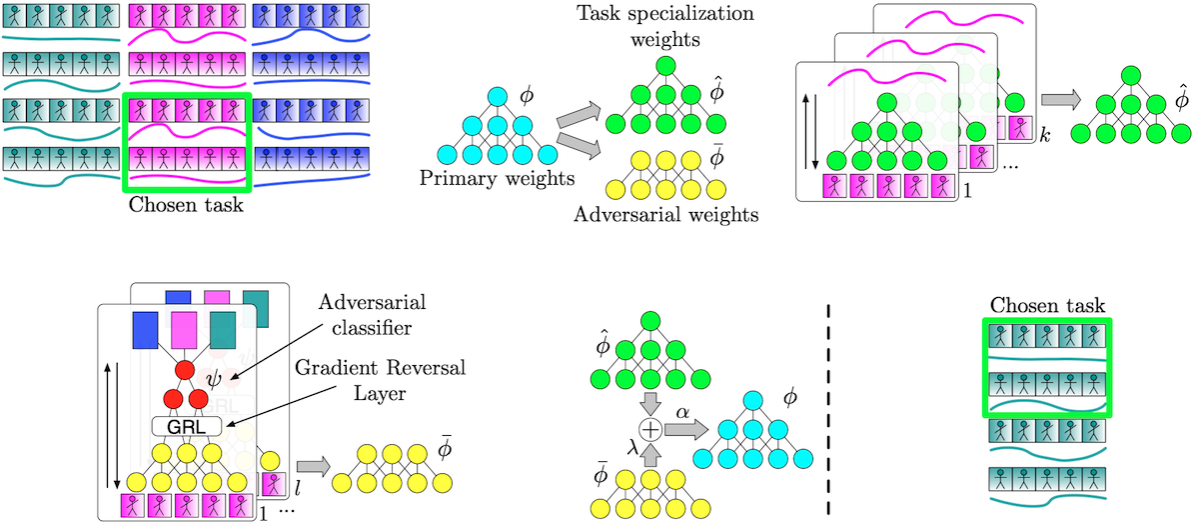
Visualization of our data pipeline used to train and fine-tune a neural network, which is then used to provide personalized energy expenditure estimations from video.

Instead of optimizing the estimation of the current network, the training process optimizes the estimation of the network after it has been fine-tuned to a random participant from the training set, while an adversarial component aims to make the initialization agnostic to the participants in the training set. Figure 2 provides an illustration of this process. Specifically, it shows that first, a small “task” is constructed from the training set, containing a small amount of silhouette video and associated calorimeter readings. Subsequently, 2 copies of the network initialization (ie, primary weights) are taken, which are named the task specialization and adversarial weights. The task specialization network is fine-tuned for a small number of iterations and becomes well suited to the current task. The adversarial weights are combined with an adversarial classifier, which are trained to predict which participant is used for this specific task. However, during this part, the gradients between the adversarial classifier and adversarial weights are negated. This means that as the adversarial classifier becomes better at classifying the person, the adversarial weights lose the ability to classify the person (ie, they become person agnostic). The task specialization and adversarial weights are finally merged back into the primary set of weights, and the process repeats with a different task. This process results in a set of primary weights that are agnostic to the participants in the training set yet are well suited to fine-tuning to unseen participants (Figure 2). For evaluation on an unseen participant, the primary set of weights are fine-tuned using a small amount of data from the unseen participant, and the adversarial component is not required because we want the evaluation network to be personalized to the evaluation participant.

## Results

### Overview

In this section, we outline our experiments and their results. Our aim is to find a network fine-tuning procedure that requires the minimum amount of data. In practice, this means less participant, clinician, and calorimeter time is required to personalize an energy expenditure model.

### Experimental Setup

A leave-one-out cross validation is used. In other words, 9 participants are used to train the model, and the 10th is used for evaluation. This process is repeated for each participant. To provide context to our results, we compare them against the following baselines: (1) MET value, which is calculated using expert labelled action start and end times; (2) no fine-tuning, which is a general model baseline as it can only make estimations with information learned from participants not being evaluated on; (3) fine-tune on one whole sequence of the participant the model is being personalized to—here, much more data are available to fine-tune than for the rest of our experiments, so this represents an upper bound for performance. The average length of a sequence is 30 minutes; (4) comparison with the work that introduces the meta-learning method in this paper [30], but only fine-tuned on the start of a sequence that contains frames without action labels; and (5) fine-tune using data from all 11 actions (32 seconds per action). This shows that standard training or fine-tuning fails with small amounts of data, even if fine-tuned with examples from all actions.

Mean square error (MSE) of the neural network estimation against the ground truth calorimeter reading is used as the evaluation metric. Note that an error is calculated for every video frame (but the model will have seen the previous 30 seconds of video to make this prediction).

There are 2 long (20-30 minutes) sequences per participant. For all experiments, the network is fine-tuned using data from sequence 1 and evaluated on sequence 2 and vice versa. This ensures that no data for evaluation have been seen during training or fine-tuning.

### Single-Action Personalization

To fine-tune to the participant being used for evaluation, 60 video clips are used. As we are assessing how well the model personalizes using a single action, these 60 clips are taken from a 32-second block of video where the fine-tuning action first appears. Each clip contains 30 uniformly sampled frames from 30 seconds of video (ie, sampling 1 frame every second). Given 32 seconds of video at 30 frames per second, there are 32*30=960 frames. The first video clip uses fames 1, 31, …, 901. The second video clip uses frames 2, 32,…, 902, and so on.

The first row of Table 1 shows the results of fine-tuning on each action compared against the baselines listed above. We can see that 32 seconds of *wipe* is best for learning a personalized calorific profile. However, it is still short of the upper bound on performance. The model fine-tuned on a whole video sequence has an MSE of 1.06 compared to 1.40 for *wipe*.

**Table 1:** Mean square error averaged across all participants.

Actions			Stand		Sit		Walk		Wipe		Vacuum		Sweep		Lie		Exercise		Stretch		Clean		Read
Single action			2.32		2.20		2.80		1.40^a^		1.94		2.15		2.43		6.77		17.85		3.27		2.47
**Action pairs**																							
	Stand	2.55		—^b^		—		—		—		—		—		—		—		—		—	
	Sit	2.65		2.09		—		—		—		—		—		—		—		—		—	
	Walk	2.87		2.57		2.72		—		—		—		—		—		—		—		—	
	Wipe	1.52		1.50		1.72		1.55		—		—		—		—		—		—		—	
	Vacuum	1.40		1.77		1.74		1.34		2.01		—		—		—		—		—		—	
	Sweep	1.61		1.09^c^		1.59		1.36		1.60		2.55		—		—		—		—		—	
	Lie	1.56		1.24		2.39		1.38		2.12		2.60		2.34		—		—		—		—	
	Exercise	2.42		1.87		2.82		3.18		2.89		2.62		3.50		5.72		—		—		—	
	Stretch	17.70		3.01		4.47		6.34		4.88		4.83		11.79		7.63		12.65		—		—	
	Clean	1.45		1.59		1.71		1.52		2.46		2.03		2.08		5.28		8.06		3.42		—	
	Read	1.98		4.98		2.47		1.40		2.16		2.24		2.44		3.43		3.02		2.42		2.35	
**Baselines**																							
	MET^d^	2.87		—		—		—		—		—		—		—		—		—		—	
	Before train only	2.17		—		—		—		—		—		—		—		—		—		—	
	All actions (whole sequence)	1.06^e^		—		—		—		—		—		—		—		—		—		—	
	All actions (32s/action)	3.30		—		—		—		—		—		—		—		—		—		—	
	Sequence start [30]	1.74		—		—		—		—		—		—		—		—		—		—	

^a^Best single action.

^b^Not applicable.

^c^Best paired action.

^d^MET: metabolic equivalent task.

^e^Best baseline.

### Multiple Action Personalization

**Figure 3:**
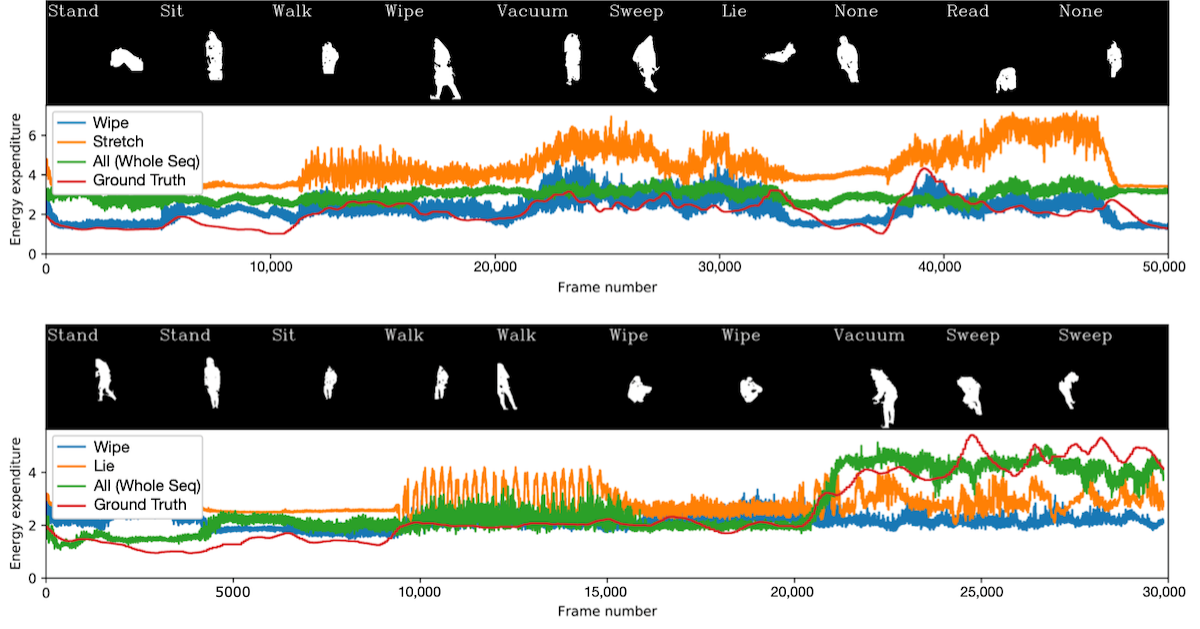
Example energy expenditure estimations from silhouettes (recorded at 30 frames per second) using single action fine-tuning. The top example shows a success case where a model fine-tuned using only 32 seconds of wipe outperforms the whole sequence baseline, and that stretch is not a good action to use. The bottom example shows a failure case, where the models fine-tuned on a single action do not adapt to the period of high energy expenditure toward the end of a sequence. Seq: sequence.

With the hypothesis that a broader range of actions provides a better calorific profiling of a person, we deploy multiple actions to fine-tune. This is motivated by the example in Figure 3 and the associated single-action personalization results, where fine-tuning on a whole sequence outperforms models fine-tuned on any single action. For the following experiments, we compare every pair of actions. For each action, the same amount of footage is available to fine-tune as there was in the previous experiments (ie, 32 seconds). Table 1 also shows the results of all 2-action combinations averaged per participant. To verify that any improvement is not just due to an increase in fine-tuning data (ie, 64 seconds from 2 actions compared to 32 seconds from 1), we include single-action results with the larger 64 seconds of fine-tuning data.

The best performing pair (*sweep* and *sit*) has an MSE of 1.09, which outperforms the best single-action pair (*wipe*, MSE 1.40). It is also very close to the whole sequence baseline, despite using much less data (64 seconds compared to 30 minutes).

**Figure 4:**
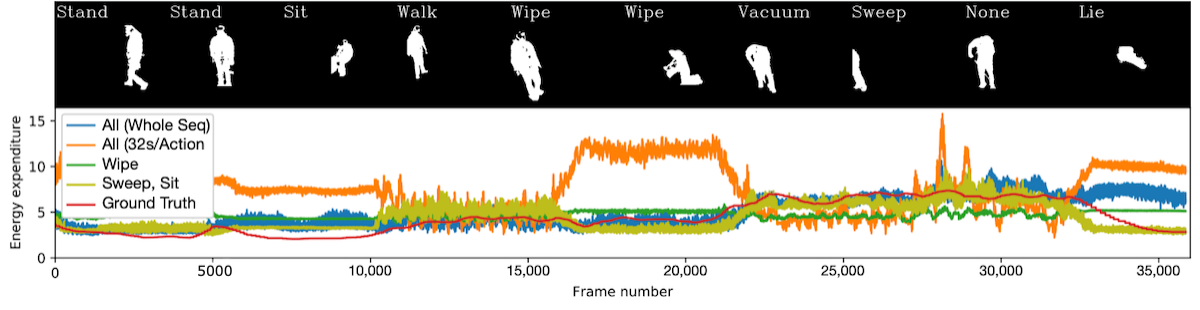
An example sequence of silhouettes and energy expenditure estimations. Here, the best pair of actions for calibration across all participants is compared against the best single action, a whole video sequence to calibrate, and shorter footage from every action. Seq: sequence.

An example of multiple-action fine-tuning is given in Figure 4, for which the whole sequence model performs the worst. The best single (*wipe*) and pair (*sweep* and *sit*) fine-tuned models are shown alongside models fine-tuned on the whole sequence and with all actions (32 seconds per action).

Finally, Table 2 details the baselines, single-action results, and selected double-action results for each person individually.

**Table 2:** Mean square error of baselines and single- and selected double-action fine-tuned models. The results are shown for each participant (“Pn”) individually along with the average over all participants. A blank entry indicates the action was not in video sequence used for fine-tuning.

Actions			Participants																					Average
			P1		P2		P3		P4		P5		P6		P7		P8		P9		P10			
**Baselines**																								
	MET^a^	2.19		2.56		1.76		0.22		2.52		3.96		8.81		1.72		1.84		3.14		2.87		
	Before train only	1.38		0.82		0.87		0.69		1.46		3.55		7.41		0.71		1.43		3.34		2.17		
	All (whole sequence)	0.60		0.54		0.62		0.14		1.54		1.54		1.75		0.28		0.55		2.02		1.06^b^		
	All (32s/action)	0.85		0.41		0.74		0.09		1.11		2.53		22.79		0.79		0.63		3.10		3.30		
	Sequence start [30]	0.29		0.58		0.54		0.29		1.25		2.30		3.24		3.50		0.65		4.73		1.74		
**Single action**																								
	Stand	0.53		0.67		0.60		0.50		1.10		5.04		4.26		2.20		0.59		7.66		2.32		
	Sit	0.49		0.92		0.42		0.21		1.13		3.02		3.35		3.12		0.42		8.96		2.20		
	Walk	0.80		0.53		0.47		0.29		2.07		7.78		4.32		2.28		0.47		8.97		2.80		
	Wipe	0.29		1.36		0.45		0.36		0.73		3.37		2.95		1.80		0.48		2.17		1.40^c^		
	Vacuum	0.79		0.63		0.54		0.60		1.67		2.95		5.18		1.89		0.85		4.29		1.94		
	Sweep	1.01		0.57		0.81		0.47		0.62		2.85		9.24		3.29		0.39		2.31		2.15		
	Lie	1.52		0.70		1.14		1.29		0.92		3.04		10.59		1.53		1.35		2.22		2.43		
	Exercise	1.56		0.76		—^d^		2.96		0.59		5.74		7.59		7.47		0.80		33.41		6.77		
	Stretch	5.93		46.52		0.48		5.82		5.16		21.19		30.64		13.86		30.81		18.05		17.85		
	Clean	1.17		2.15		0.94		0.32		1.04		5.93		8.94		2.17		4.65		5.35		3.27		
	Read	2.05		1.35		0.84		0.56		0.81		2.53		7.50		1.92		2.22		4.90		2.47		
**Action pairs**																								
	Sweep/sit	0.96		0.67		0.47		0.13		0.90		2.51		1.02		0.99		0.47		2.75		1.09^e^		
	Lie/sit	0.61		0.53		0.43		0.45		0.82		2.69		3.00		1.07		0.60		2.24		1.24		
	Vacuum/stand	0.87		0.57		0.38		0.14		1.53		3.39		1.73		0.78		0.61		4.04		1.40		
	Vacuum/wipe	0.48		0.64		0.64		0.19		1.21		3.25		1.59		1.63		0.60		3.15		1.34		
	Sweep/wipe	0.60		0.59		0.67		0.16		1.01		2.52		4.16		1.02		0.54		2.35		1.36		
	Wipe/wipe	0.57		0.95		0.48		0.11		1.07		3.88		3.43		1.98		0.56		2.42		1.55		
	Stretch/exercise	2.83		2.19		—		4.18		3.58		8.74		14.63		8.93		0.93		22.70		7.63		
	Clean/stretch	5.01		5.39		0.57		2.20		2.79		11.90		21.57		7.26		17.12		6.80		8.06		
	Stretch/lie	1.78		9.14		0.61		1.19		3.70		2.98		77.20		3.46		9.64		8.23		11.79		
	Stretch/stand	1.72		2.63		0.56		1.72		1.96		8.08		146.70		5.08		3.49		5.01		17.70		

^a^MET: metabolic equivalent task.

^b^Best baseline.

^c^Best single action.

^d^Blank entries indicate the action was not in the video sequence used for fine-tuning.

^e^Best action pair.

## Discussion

### Single or Pair Difference

The results presented above raise several points for discussion. Perhaps the most important is why the best single action to fine-tune with (*wipe*) is not part of the best pair to fine-tune with (*sweep* and *sit*). Given a distribution of calorimeter or silhouette sequences (which contain a wide variety of actions and calorific profiles), we would expect fine-tuning with 1 action to cover the middle of this distribution. If 2 actions are available, then each can be representative of more extreme parts of the energy expenditure or silhouette distribution while still adequately covering the middle of the distribution; 2 actions outperforming 1 corresponds to this intuition

### Action Variation

Another interesting observation is that there is a large amount of variation when fine-tuning using different actions. For example, fine-tuning using *stretch* is much worse than any other single action (17.85 MSE compared to the baseline 1.06 and second worst 6.77). One possible reason is that a participant stretching produces very different silhouettes compared to any of the other actions they perform. If a model is fine-tuned using these silhouettes, it has been conditioned to very different data compared to the other actions and thus gives bad estimations. A similar effect can be seen with *exercise*, which has less extreme but different silhouettes (6.77 MSE). This reasoning also applies to specific actions outperforming models fine-tuned on the sequence start. The sequence start may not provide enough information about a participant’s calorific profile for the fine-tuned model to work well across a wide variety of actions.

### Participant Variation

There is also a difference in how all methods perform on specific participants. In particular, all models struggle on P10, with even the model fine-tuned on a whole sequence giving an MSE of 2.02. This is unlikely to be caused by visual differences (in the way that models fine-tuned on *stretch* are) as all actions perform poorly. Rather, it is most likely due to P10 having a calorific profile, which is very dissimilar to those found in all the other participants and could possibly be remedied by collecting data from more participants to use during the training of the initialization.

### Conclusion

In this paper, we showed that a personalized calorie expenditure model that is more accurate than other existing techniques (bar intrusive calorimetry devices) is possible using a vision-based deep learning technique. The method can be personalized and can perform indefinitely in clinical and home environments after just 64 seconds of calorimeter calibration.

Our method uses a state-of-the art deep learning technique, which learns an initialization from a data set containing calorimeter readings of footage from multiple participants. The initialization can then be adapted quickly to a participant unseen in the training set with footage and calorimeter readings of them *sweeping* for 32 seconds and *sitting* for 32 seconds. This personalized model outperforms the general models that have been used in the past.

The method outlined in this paper provides some benefits. It is suitable for long-term continuous monitoring of energy expenditure in daily-living scenarios and environments as it is noninvasive and does not require any change to participant behavior. It requires very little expensive clinician and calorimeter time to personalize, and it only needs a relatively cheap RGB-D sensor. Further, it does not require any human annotation of actions or activities after recording has finished.

## Abbreviations

MET: metabolic equivalent task
MSE: mean square error
RGB-D: Red, Green, Blue plus Depth
SPHERE: Sensor Platform for Healthcare in a Residential Environment
